# Origins of Functional Organization in the Visual Cortex

**DOI:** 10.3389/fnsys.2020.00010

**Published:** 2020-03-03

**Authors:** Michael Ibbotson, Young Jun Jung

**Affiliations:** ^1^Australian College of Optometry, National Vision Research Institute, Carlton, VIC, Australia; ^2^Department of Optometry and Vision Science, The University of Melbourne, Parkville, VIC, Australia

**Keywords:** primary visual cortex (V1), orientation selectivity, cortical maps, retinotopy, phylogeny, visual system

## Abstract

How are the complex maps for orientation selectivity (OS) created in the primary visual cortex (V1)? Rodents and rabbits have a random distribution of OS preferences across V1 while in cats, ferrets, and all primates cells with similar OS preferences cluster together into relatively wide cortical columns. Given other clear similarities in the organization of the visual pathways, why is it that maps coding OS preferences are so radically different? Prominent models have been created of cortical OS mapping that incorporate Hebbian plasticity, intracortical interactions, and the properties of growing axons. However, these models suggest that the maps arise primarily through intracortical interactions. Here we focus on several other features of the visual system and brain that may influence V1 structure. These are: eye divergence, the total number of cells in V1, the thalamocortical networks, the topography of the retina and phylogeny. We outline the evidence for and against these factors contributing to map formation. One promising theory is that the central-to-peripheral ratio (CP ratio) of retinal cell density can be used to predict whether or not a species has pinwheel maps. Animals with high CP ratios (>7) have orientation columns while those with low CP ratios (<4) have random OS maps. The CP ratio is related to the total number of cells in cortex, which also appears to be a reasonable contributing factor. However, while these factors correlate with map structure to some extent, there is a gray area where certain species do not fit elegantly into the theory. A problem with the existing literature is that OS maps have been investigated in only a small number of mammals, from a small fraction of the mammalian phylogenetic tree. We suggest four species (agouti, fruit bat, sheep, and wallaby) that have a range of interesting characteristics, which sit at intermediate locations between primates and rodents, that make them good targets for filling in the missing gaps in the literature. We make predictions about the map structures of these species based on the organization of their brains and visual systems and, in doing so, set possible paths for future research.

## Introduction

**FIGURE 1:**
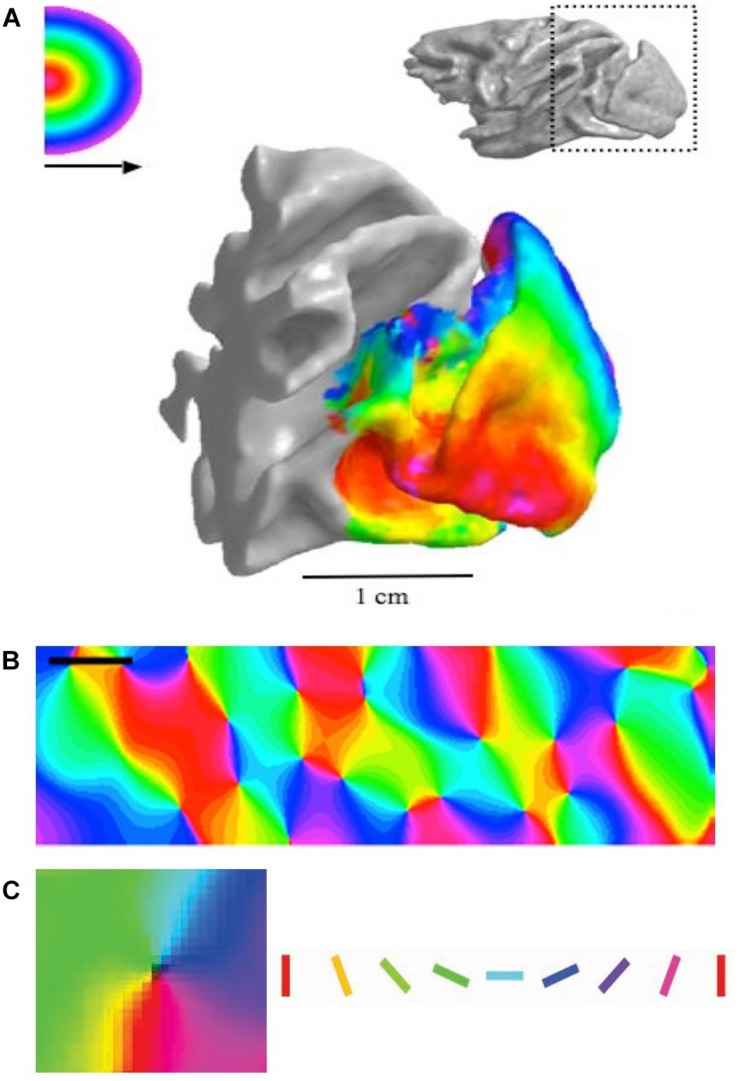
**(A)** Retinotopic map in monkey visual cortex (fMRI data). **(B)** Orientation map in cat cortex measured using optical imaging, with orientation selectivity coded by specific colors. Scale = 1 mm. **(C)** Zoom onto a single pinwheel in cat cortex. Square width = 1 mm. Oriented bars show color code in panels **B** and **C**. Panel **A** reproduced from Brewer et al. (2002) with permission from the copyright holder. Panels **B** and **C** adapted from Cloherty et al. (2016) with permission from the copyright holder.

The cerebral cortex is common to all mammals. While different regions of the cerebral cortex specialize in processing disparate signals, e.g. sensory coding, motor output, decision making, it is striking that the fundamental structure of the cortex is conserved across these regions. Anatomically, almost all regions of the mammalian cortex are composed of six distinct neuronal layers with a common set of neuronal circuit elements (Briggs, 2010; Seelke et al., 2012; Markram et al., 2015). Functionally, cortical architecture is structured similarly between brain regions, each possessing systematic maps of the features they code: e.g. retinotopy in visual areas (Figure 1A; Brewer et al., 2002), tonotopy in auditory areas (Pantev et al., 1995), and homunculi (maps of the body) in somatosensory and motor areas (Feldman and Brecht, 2005; Harrison et al., 2012). *It is generally believed that these patterned maps are an integral part of the computations performed by the cortical circuits.*

Comparative approaches have proved very useful for identifying genetically driven distinctions in the functional organization of mammalian nervous systems. For example, the corpus callosum is not present in monotremes and marsupials (metatherians) but is found in placental (eutherian) mammals, providing a timeline for the emergence of this feature (reviewed in Kaas, 2017). While this article reviews what is currently known, the relative lack of data in some areas has led us, here, to take an expanded approach – we propose several theories and experiments that would greatly assist in creating a better understanding of map structures in cortex. The rationale for such experiments is placed into the context of the reviewed literature.

### Cortical Maps

In every mammalian species studied, V1 has a retinotopic map in which information from the visual field is coded onto a two-dimensional surface that retains the image’s spatial organization. Embedded in the retinotopic map, V1 also codes other stimulus features, e.g. the orientation of edges, the eye of origin (ocular dominance), spatial frequency, and the direction of motion (Goodhill, 2007). There is an extensive literature describing how various stimulus features can be mapped onto the cortex, which shows that self-organizing mechanisms are good at reproducing V1 maps in species such as cats, ferrets, and primates, but they may not provide all the answers (Hubel et al., 1977; Blasdel and Salama, 1986; Bonhoeffer and Grinvald, 1991; Bartfeld and Grinvald, 1992; Obermayer and Blasdel, 1993; Hübener et al., 1997; Nauhaus et al., 2012).

Here, we will focus on the maps of orientation selectivity (OS), which need to code oriented structures in every patch of visual space through the full 180°. It has been suggested that detecting the orientation of edges in visual scenes is as fundamental to visual processing as detecting brightness (Bell and Sejnowski, 1997). Some species (e.g. primates and cats) organize their OS cells into highly structured orientation columns, where cells with the same preference cluster together into columns that are 0.2–0.7 mm in diameter (Figure 1B; e.g. Cloherty et al., 2016). In these species, all cells through all six cortical layers have the same OS, suggesting an emphasis on vertical integration of feature selectivity through the cortical layers. To ensure that all orientations are represented in every patch of the retinotopic map, the orientation columns representing different orientations are arranged radially around a central location in the horizontal plane, centered on a particular point in the retinotopic map (Figure 1C). This cortical architecture is known as a “pinwheel” OS map. Other species have a random distribution of orientation selective cells, i.e. most cells are OS but those with different orientation preferences are juxtaposed in a random fashion (rodents/rabbits: Ohki et al., 2005; Van Hooser et al., 2005; Bonin et al., 2011; Espinosa and Stryker, 2012; Reid, 2012). The cells are intermingled both horizontally across the retinotopic map and vertically through the six cortical layers. This is known as a “random” or “salt-and-pepper” OS map.

### Essential Techniques

Five essential experimental techniques need to be briefly discussed before reviewing current and future directions in this field. (1) Intrinsic optical imaging (OI) is a technique that allows cortical maps to be measured with a high degree of accuracy over relatively wide regions of the cortex down to depths of 600–1000 μm, with a horizontal resolution of ∼50 μm. This technique is simple in principle but complex in operation. A bright red or green light is shone onto the cortex and this partially penetrates into the neural tissue (Grinvald et al., 1986; for recent red–green comparison, see Cloherty et al., 2016). Areas that have large volumes of blood or increase in deoxy-hemoglobin concentration due to high levels of neural activity do not reflect much light, so they appear dark. In contrast, areas with less blood flow reflect more light, so they appear brighter. In this way, it is possible to determine which areas of cortex are active and which are not, thus allowing the measurement of cortical maps (Figures 1B,C). (2) Two photon calcium imaging allows much higher spatial resolution in three-dimensions but is restricted to far smaller areas of the cortex. This technique, for example, allows the visualization of single neurons in an entire column of cortex (Tischbirek et al., 2019). The two-photon technique requires the cells to be loaded with a calcium sensitive dye or made to express a genetically encoded calcium indicator. Both techniques allow the activity-dependent fluorescent signals to be measured, thus allowing fine detailed mapping of cortical areas. (3) Whenever investigating cortex, it is useful to record electrical responses from cortical cells at the spiking level. This not only provides essential information about neural processing but also validates the data from the OI (Zepeda et al., 2004). In recent years multi-electrode recordings have become possible and this has greatly improved the capacity to correlate OI with massed recording techniques (Nauhaus and Ringach, 2007). (4) Dye injection into various regions of brains allows the local and long-distance connectivity to be established (Bosking et al., 1997). This is fundamental to understanding cortical map structures. For example, dye injections into the lateral geniculate nucleus (LGN), which is the relay center between the retina and V1 can establish the topography of the retinal cells that provide the input to V1 (e.g. Wimborne et al., 1999). (5) Finally, computer modeling is a critical technique in understanding cortical maps in several important ways (Durbin and Mitchison, 1990; Swindale, 1996; Koulakov and Chklovskii, 2001; Goodhill, 2007). These models offer potential mechanisms that can be tested experimentally and provide quantitative methods for developing new theories.

### Understanding Cortical Maps Through Comparative Physiology

Why do some mammals have salt-and-pepper OS maps and others pinwheel OS maps? This question is a hotly debated topic in the fields of visual neuroscience and developmental neurobiology (Horton and Adams, 2005; Van Hooser et al., 2005; Kaschube et al., 2010; Scholl et al., 2013; Vidyasagar and Eysel, 2015; Kremkow et al., 2016). There are many factors that might influence evolution’s choice of OS map. These include (a) predator versus prey, (b) the degree to which animals are nocturnal, (c) the need for binocular processing based on the divergence of the eyes, (d) the size of the brain (or visual cortex), (e) the sophistication of the cortical architecture, (f) the resolution of the visual system, (g) the thalamocortical networks that provide their visual inputs, (h) the distribution of retinal ganglion cells (RGCs) in the retina, which is thought to be related to each species’ visual environment (Hughes, 1977), or (i) a genetic factor related to phylogeny.

We will begin by discounting the first two things on this list. Might predators have pinwheel structures and herbivores a salt-and-pepper structure? Primates contradict this notion as all have pinwheel maps but most are fructivores. Moreover, a carnivorous rodent has been shown to have a salt-and-pepper OS map (Scholl et al., 2017). Could OS map structure be related to nocturnal versus diurnal lifestyles? This is unlikely as cats and some primates are primarily nocturnal but have pinwheel OS maps. Also, some rodents (squirrels) are diurnal but have salt-and-pepper OS maps. We will compare several possibilities: eye divergence, the total number of cells in V1, the thalamocortical networks that provide their visual inputs, the topography of the retina and the phylogenetic relationships between mammalian species, and discuss how each factor may influence cortical map structure. We propose that the central-to-peripheral ratio (CP ratio) of retinal cell density is good at predicting the presence or absence of OS maps.

### Understanding Cortical Maps Through Eye Divergence

**FIGURE 2:**
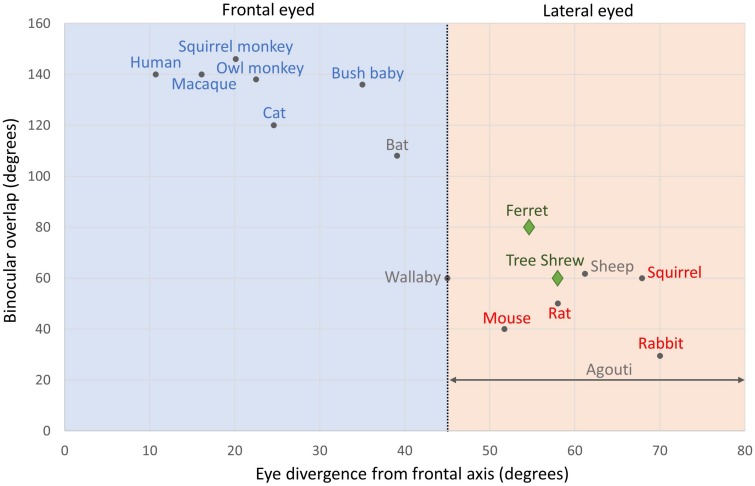
Binocular overlap plotted against eye divergence in mammals. The black vertical line shows an eye divergence of 45 degrees, which divides frontal from lateral eyed animals: Eye divergence = 90 degrees – Orbit convergence. Species names in blue have pinwheel maps. Species names in red have salt-and-pepper maps. Ferrets and tree shrews have names and symbols in green: both have pinwheel maps. Species names in gray are those that we suggest should be investigated in future studies: agouti, fruit bat, sheep, and wallaby. Adapted from Heesy (2004) with permission from the copyright holder. Binocular overlap data for agouti is from Picanço-Diniz et al. (2011).

Could animals with frontal eyes have pinwheel OS maps while lateral eyed animals have salt-and-pepper maps? Figure 2 plots binocular overlap in the visual field against eye divergence for mammals that have had their cortical OS maps assessed. Pupillary (eye) divergence can be quantified by photographing the eyes while the animal is facing a distant light source. The images of this light, formed by reflection at the corneas, coincide approximately with the optical centers of the eyes, and the distance by which the separation of the pupils exceeds the separation of these images is defined as pupillary divergence (Olson and Freeman, 1978). Figure 2 shows which species have pinwheel (blue) or salt-and-pepper (red) maps. The species in blue at the top left are from humans, macaques, squirrel monkeys, owl monkeys, bushbabies, and the domestic cat. The species in red at the bottom right are squirrels, rats, mice, and rabbits. If our analysis stopped there, we could draw quite a robust conclusion that a lack of orientation columns in the cortex is associated with animals that have highly diverged eyes and small binocular fields, while those with pinwheel structures in cortex have small eye divergence and large binocular fields. However, tree shrews and ferrets (green diamonds) contradict this simple theory because both have quite laterally positioned eyes and small binocular overlaps, yet they have exquisite pinwheel OS maps (Bosking et al., 1996; Rao et al., 1997). The species in gray in Figure 2 show four species of mammals that have not had their cortices imaged (i.e. sheep, wallaby, fruit bat, and agouti). Measuring the cortical map structures in these species would clearly help to fill in essential missing data.

The agouti clearly has quite lateral eyes but no specific optical measure has been conducted, so we show it as a span of eye divergence angles (horizontal gray line). However, its binocular overlap has been measured as 20° (Picanço-Diniz et al., 2011). The wallaby is a particularly interesting species to study as its eye divergence is almost exactly 45°, placing it between the two extremes. The bat and the sheep are also interesting species to examine as they naturally cluster on this plot, respectively, with the frontal or lateral eyed species but electrophysiological evidence suggests that the sheep might have pinwheel cortical maps (see below), while we can only hypothesize about the bat at present.

### Understanding Cortical Maps Through V1 Cell Numbers, Cortical Complexity, and Spatial Resolution

Could it be that mammals with smaller primary visual cortices, relatively undifferentiated cortices or poor-resolution vision are restricted to having salt-and-pepper OS maps? These concepts were thoroughly investigated by selecting a rodent that has good spatial resolution, and a V1 that is highly differentiated and has a surface area similar to that of animals with pinwheel OS maps; the species selected was the gray squirrel (Van Hooser et al., 2005). It was found that despite a well-developed cortex the squirrel has a salt-and-pepper map. Despite the findings from the squirrel, Harris and Mrsic-Flogel (2013) later proposed that an ordered orientation map in a physically small cortex would lead to poorer coverage of orientations in their visual field than a random structure. However, this suggestion breaks down somewhat when comparing tree shrews and rabbits. The area of V1 is in fact smaller in tree shrews (60 mm^2^) (Muly and Fitzpatrick, 1992) than rabbits (80 mm^2^) (Hughes, 1971) but tree shrews have orientation columns while they are absent in rabbits.

**FIGURE 3:**
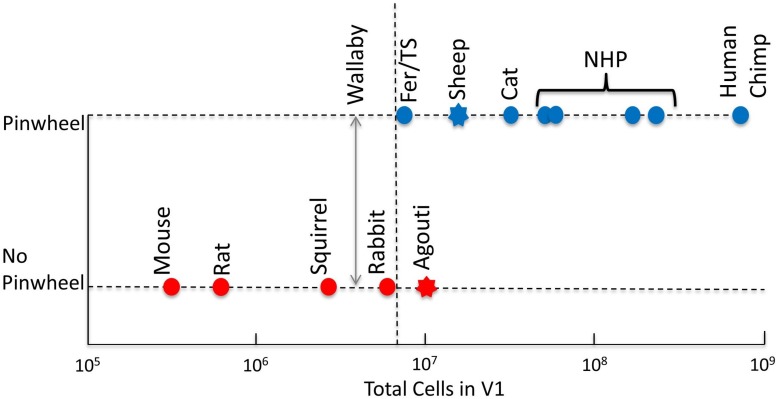
Existence of pinwheels plotted against the number of cells in V1. Red, no pinwheels; blue, pinwheels. Circles show results from intrinsic optical imaging. Stars show results obtained only from single-cell recording. The dotted vertical line shows the possible threshold between species with pinwheels and those without. Note that the Agouti has more cells in V1 than both ferrets and tree shrews but the indication is that they have a salt-and-pepper map. The double-headed vertical arrow shows the cell count in wallaby V1 – it is not yet known if they have pinwheels. Fer, ferret; TS, tree shrew; NHP, non-human primates (including marmoset, bush baby, owl monkey, squirrel monkey, macaque); Chimp, chimpanzee. Reproduced from Weigand et al. (2017) with permission from the copyright holder.

More recently, Weigand et al. (2017) reactivated the discussion about the relationship between the size of V1 and the type of OS map structure. They presented a model in which the number of cells in V1 influences the interactions between neurons – according to their model, the more cells the more likely the brain organizes itself into columns. To support this notion, they presented a graph that plotted cell number in cortex against the existence, or not, of OS maps. We present a modified and updated version of that plot in Figure 3. It shows that all primates and the cat have large cortical cell populations (>30 M) and pinwheel maps, while all rodents that have had their OS maps studied have low numbers of cortical cells (<3 M) and salt-and-pepper maps. However, it is species that fall between these extremes that create a problem for their “more cells more complexity” theory. The rabbit has nearly 6 M cells in its V1 and a clear salt-and-pepper design. The ferret and tree shrew have, respectively, 7.6 and 8 M cells, which is not dissimilar to rabbits. If only the number of cells dictates the map structure in visual cortex, the threshold must be finely balanced between 6 (rabbit) and 7.6 M cells (ferret).

Unfortunately, other species that Weigand et al. (2017) highlight have not had their orientation map structures examined using OI (stars, Figure 3). For example, the agouti is a large rodent that has 12 M cells in visual cortex. While no OI has been done, a correlation of orientation preferences based on distance across the cortex has been conducted for the agouti (Ferreiro, 2018). It showed that the local correlations in OS were similar to those of the mouse and very different to the cat. In the cat the similarity of orientation preferences changes slowly and smoothly, which corresponds with a pinwheel map structure. These smooth changes did not occur in the agouti. Instead there were abrupt changes in the OS of neighboring cells with a clustering of cells with similar orientation preferences occurring only over very short ranges. Therefore, the agouti may at most have minicolumns, which would appear with two-photon calcium imaging, as is the case in mice (Kondo et al., 2016). This mapping does not support Weigand et al.’s (2017) theory (because ferret and tree shrew have far smaller cell numbers in cortex) but does support the theory that all rodents have a consistent lack of the classic pinwheel OS maps, as found in other mammals (see the section “Understanding Cortical Maps Through Phylogeny”). Given Ferreiro’s results, we have tentatively placed the agouti on the “no pinwheel” line in Figure 3.

The sheep has 18 M cells in cortex, but its OS map structure remains unknown. Based on the observation that orientation preferences changed slowly during single electrode recordings it is likely that the sheep has a structured, pinwheel-like orientation map (Clarke et al., 1976; Ramachandran et al., 1977; Kennedy et al., 1983). Therefore, we have tentatively placed the sheep on the “has pinwheel” line in Figure 3. Clearly, an imaging investigation of agouti and sheep cortices would be a very useful way of filling in the missing data to confirm or reject the theory of Weigand et al. (2017). Rather than thinking of the data confirming or rejecting the theory, it is also worth considering that the theory has merit but in the transition zone between large and small numbers of cortical cells, other factors also play important roles, as outlined below. Unfortunately, we could not add the fruit bat into Figure 3 because we do not yet have a reliable measure of the number of cells in fruit bat V1.

### Understanding Cortical Maps Through Understanding the Visual Pathways

Recent work suggests that cortical maps are seeded by the thalamocortical networks that provide their input (Paik and Ringach, 2011; Nauhaus and Nielsen, 2014; Wang et al., 2015; Kremkow et al., 2016; Lee et al., 2016). The visual pathway to the cortex is from RGCs to LGN to V1. Each V1 neuron receives input from a bundle of thalamic afferents that control the shape of the cortical receptive field. The ON and OFF thalamic afferents rotate around each other to minimize the cortical wiring needed to represent visual points (Schummers et al., 2002). It has been revealed through experimental studies that the receptive field structure of ON and OFF thalamic afferents can predict orientation preference and the clustering of cells in cortical domains (Koch et al., 2016; Kremkow et al., 2016). However, it remains unknown whether ON–OFF rotation in receptive field position is developed by the mosaic arrangement of ON and OFF ganglion cells in retina (Wässle et al., 1981; Soodak et al., 1987; Ringach, 2004; Paik and Ringach, 2011) or by the strongly correlated firing between ON and OFF thalamic afferents (Miller, 1994; Goodhill and Löwel, 1995; Nakagama et al., 2000).

A theory proposed by Paik and Ringach (2011) suggests that Moire interference patterns formed by the ON and OFF ganglion cell arrays create different patterns of OS in the cortex, which resemble pinwheel maps. If true, this model could have a role in determining whether a species has pinwheel maps. However, the model requires that the ON and OFF arrays are geometrically precise while the system is imprecise in lining up the two arrays. Hore et al. (2012) showed that the RGCs do not have the spatial distributions required to create perfect ON and OFF retinal mosaics. Additionally, a more recent computational model by Schottdorf et al. (2014) suggests that the spatial structure of cat and macaque retinal mosaics are not plausible for seeding the orientation preference maps of the primary visual cortex. It is also not clear how the geometrical realities imposed by different retinal designs might affect the arrangement of ON and OFF RGCs (see below for retinal designs).

**FIGURE 4:**
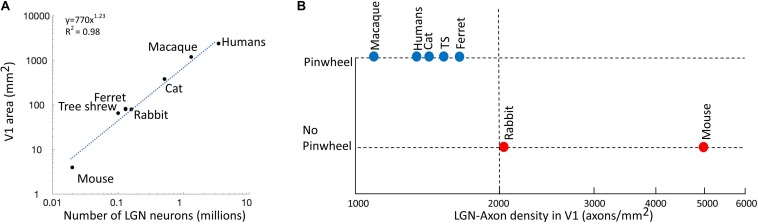
The LGN-axon densities in V1. **(A)** The number of LGN cells is correlated to the size of area V1. Blue dotted line shows an exponential plot (*y* = 770*x*^1.23^, *R*^2^ = 0.98). **(B)** Existence of pinwheels plotted against the LGN-axon density in V1. Red, no pinwheels; blue, pinwheels. The dotted vertical line shows the possible threshold between species with pinwheels and those without. Reproduced from Mazade and Alonso (2017) with permission from the copyright holder.

Mazade and Alonso (2017) propose that the spacing between thalamic axon patches with overlapping receptive fields needs to be greater than 2 axon patches to allow afferents to cluster within different cortical domains. As shown in Figure 4A, as the number of LGN neurons increases, the size of V1 increases in a non-linear fashion, as shown by the exponential plot (*y* = 770*x*^1.23^, *R*^2^ = 0.98) (Stevens, 2001), i.e. V1’s size becomes much larger than that expected from a linear co-scaling of thalamus and cortex. In primates, carnivores, and scandentia (e.g. tree shrew), the overexpansion of area V1 makes the density of LGN axons per mm^2^ of cortical area smaller. In Figure 4B, we can see that species with low densities of LGN axons in V1 have pinwheel maps, while in species with high LGN densities (>2000 axons/mm^2^) the pinwheels are absent. It seems that there is a certain threshold between species with pinwheels and those without, which we have tentatively drawn as a dashed line at 2000 axons/mm^2^. Unfortunately, we do not have information regarding the LGN densities of the four species that we have highlighted above as future candidates, making such studies worthwhile future pursuits.

The high total number of LGN neurons in primates and carnivores are associated with larger numbers of central RGCs (Kremkow and Alonso, 2018). As a result of evolutionary pressure, primates and carnivores have greater binocular overlap and in turn, there is a greater percentage of RGCs projecting to the LGN. In primates and cats, most RGCs project to the LGN and very few project to other subcortical regions (Illing and Wässle, 1981; Perry and Cowey, 1984). In comparison, most of the mouse and rabbit RGCs project to the superior colliculus and other subcortical regions responsible for visual navigation (Ellis et al., 2016).

Different mammalian species have different ways of organizing their retinal inputs to accommodate their visual needs. For example, the retinal inputs in rabbits are devoted to peripheral vision and offer a panoramic view of the environment. This gives them the best opportunity to lookout for predators (Oyster et al., 1981). On the other hand, primates have a strong retinal bias toward central vision to process high acuity vision. Therefore, the spatial distribution of retinal inputs is an important factor determining the number of thalamic afferents to V1 (see below for more on retinal topography).

### Understanding Cortical Maps Through Retinal Topography

**FIGURE 5:**
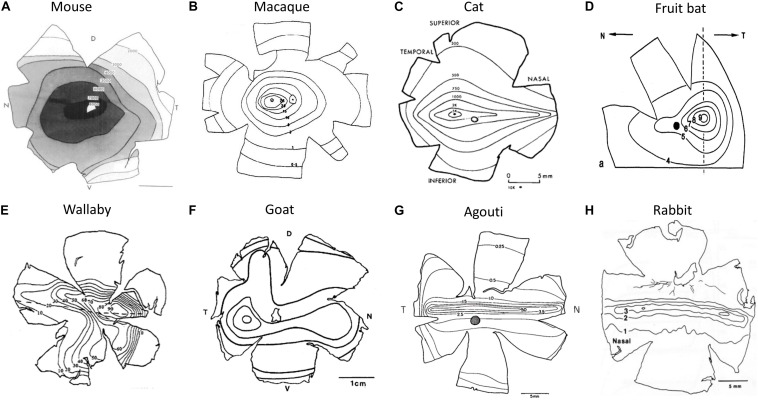
Retinal cell isodensity contour maps. **(A)** Mouse retina, **(B)** rhesus macaque retina, **(C)** cat retina, **(D)** bat retina, **(E)** wallaby retina, **(F)** goat retina, **(G)** agouti, and **(H)** rabbit. Parts **A** reproduced from Dräger and Olsen (1981), **B** from Perry and Cowey (1985), **C** from Hughes (1975), **D** from Pettigrew (1986), **E** from Wimborne et al. (1999), **F** from Gonzalez-Soriano et al. (1997), **G** from Silveira et al. (1989), and **H** from Oyster et al. (1981) with permission from the copyright holder.

Here we use a centroperipheral ratio, CP, which is the ganglion cell density in the center of the retinal specialization divided by the peripheral cell density (Navarro-Sempere et al., 2018). We propose that the CP ratio is high among species with organized pinwheel maps and low in those without. Moreover, the CP ratios correlate strongly with pinwheel density (per mm^2^) in species with pinwheel maps. To show this, we have conducted a comparative analysis of ganglion cell distribution maps across various species for which visual cortex has been studied. Each species has a unique arrangement of retinal specializations (areas of higher cell density), which appears to be influenced by ecological and developmental selective pressures in its ecological niche and habitat (Hughes, 1977; Collin, 1999, 2008). Mammalian retinas have been traditionally divided into two types: those with an *area centralis* (AC) and those with a visual streak (VS) (Figure 5; Moore et al., 2012). However, this terminology does not tell the whole story. A quantitative measure such as the CP factor may help us understand in more detail how RGC organization influences V1 organization.

Published retinal topography maps have iso-density contour lines showing variations in cell density across the retina. The iso-density lines are used to measure the changes in cell density from retinal periphery to the retinal specialization (Moore et al., 2012). The center of retinal specialization is the point with the highest RGC density, which is often marked as a black dot in the topographic maps. If this point was not reported in published maps, the center of retinal specialization density was determined as the density marked on the inner most iso-contour line of the map. The peripheral density was defined as the density at the extreme edge of the retina (in nasal and temporal regions). When the outer perimeter of the retina was not available, the cell density of the first iso-density line shown nearest the outer perimeter of the retina was halved. This method was utilized by Moore et al. (2012), based on the patterns observed in the topographic maps that included information on the outer perimeter of the retina. In most species, the cell densities of nasal and temporal periphery were the same, but for mouse, cat, sheep, and agouti, they were different. So, the nasal and temporal peripheral values were averaged for these species. We assumed that the retinal whole-mount methodology was similar across studies to produce the topographic maps. For instance, we assumed that the wholemount was correctly oriented and the degree of shrinkage was similar across studies. Also, we assumed that the cells counted were all RGCs, because in some studies the amacrine cells were difficult to distinguish from the ganglion cell layers (Hughes, 1975; Vitek et al., 1985), thus, potentially resulting in higher cell densities in the periphery. We understand that the assumptions mentioned above could introduce a certain degree of error in our measurements. Despite these limitations, we believe the CP ratios can be used to study how retinal topography influences cortical OS maps.

Arboreal species or those inhabiting dense forests generally have an AC, i.e. cell isodensity contours are circular and are centered roughly on the middle of the retina (e.g. Figures 5A–C). This type of design provides a range of acuities across the visual field in all directions. However, not all retinas with an AC are the same. Three general types of AC have been identified (Hughes, 1977). Some species with an AC have low ganglion cell densities with little centroperipheral gradient (CP ratio < 3.0). This is common in small nocturnal animals living in scrubland, such as mice, rats, and guinea pigs (Figure 5A). Others have moderate ganglion cell densities but the centroperipheral density gradient remains low (<4.0). This characteristic is predominant in small diurnal animals with relatively high visual acuity (e.g. squirrels). Finally, there are species with very large centroperipheral density gradients (>30). This final category, which is most often associated with primates, offers very high visual acuity in the central visual field (Figure 5B and Table 1).

**TABLE 1:** Relationship between retinal ganglion cell arrangement and orientation map design.

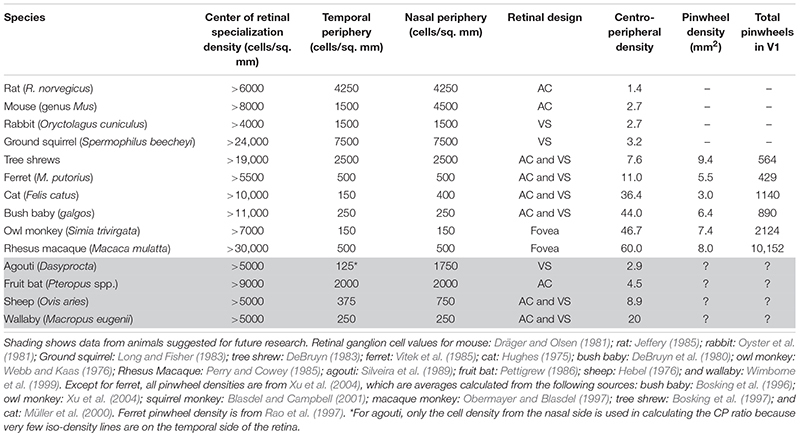

Instead of circular isodensity contours in the retina, many species have an elongated, horizontal region of the retina that has relatively high cell density, referred to as a VS. However, there are two types of VS design. Some mammals have a VS in which cell density is consistent along the entire horizon, such as rabbits and agouti (Figures 5G,H). This design is usually associated with prey species that have lateral eyes. Such a design offers a panoramic view of the environment without having to make horizontal head movements (Hughes, 1977). The second type of VS occurs in a wide variety of mammals. In this case there is a horizontal streak but the region of the retina that points directly forward has a zone of particularly high cell density (Figures 5D–F). In cats the adult eye divergence from the frontal axis is around 8 degrees (Olson and Freeman, 1978). The region of higher density in their VS is concomitantly displaced laterally by around 8 degrees in each eye to maximize visual acuity frontally. In the case of the cat, most authors state that they have an AC but in reality they have a combination of a VS and an AC. Similarly, in the sheep/goat retina, the lateral region of the VS has very high cell density at the point associated with frontal vision (Clarke and Whitteridge, 1976; Hebel, 1976). We do not have a contour map from the sheep but instead provide one from the goat retina (Figure 5F). Indeed, while sheep/goats are usually said to have a VS, the displaced region of high cell density has higher cell counts than the central region of the cat eye. Many herbivores have this combination of a VS and a lateral region of high cell density, e.g. goats, horses, and red kangaroos (Hughes, 1977). However, many carnivores also have this retinal arrangement, e.g. dogs and cats (McGreevy et al., 2004).

**FIGURE 6:**
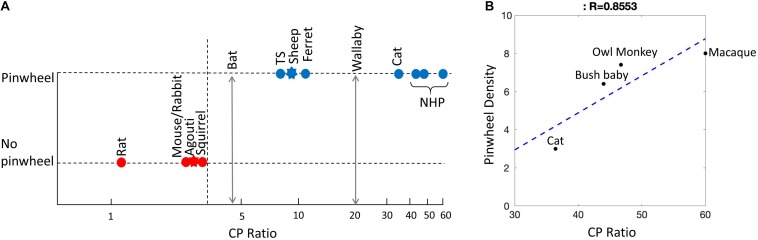
Relationship between retinal ganglion cell arrangement and orientation map design. **(A)** Existence of pinwheels plotted against the CP ratios. Red, no pinwheels; blue, pinwheels. Circles show results from intrinsic optical imaging. Stars show results obtained only from single-cell recording. The dotted vertical arrow shows the possible threshold between species with pinwheels and those without. The double-headed vertical arrows show wallaby and bat CP ratios – it is not yet known if they have pinwheels. TS, tree shrew; NHP, non-human primates (including bush baby, owl monkey, macaque). **(B)** For cat and primates, the pinwheel density (per mm^2^) is plotted as a function of CP ratio. The linear regression is shown as a blue dashed line (*R* = 0.8553).

The CP ratio varies significantly across species. Based on the data in Figure 6A and Table 1, we can see that for species without a pinwheel map (e.g. rat, gray squirrel, mouse, and rabbit), their CP ratios are all <4 (Figure 6A). That is, the central density is never more than four times that of the periphery. The agouti also has a low CP ratio due to its rabbit-like VS (red star, Figure 6A). Evidence suggests that the agouti may have a salt-and-pepper OS map, so it should sit in the left side of Figure 6A with all the other rodents. In primates and cats we can see a positive, linear correlation between CP ratios and pinwheel density (Figure 6B). Macaque monkeys have a high CP ratio of 60 and the cortical pinwheel maps are dense (8 pinwheels per mm^2^). In bushbabies and owl monkeys the CP ratios are 44 and 47 and the respective pinwheel densities 6.4 and 7.4 (Table 1). In the cat, where the displaced AC is prominent and has a high-density gradient close to primates (CP ratio of 36), the orientation columns are unexpectedly broad (3 pinwheels per mm^2^) (Figure 6).

Tree shrews and ferrets have moderate CPs and they have orientation columns but they seem to be intermediate between species that have pinwheels and those without. In the ferret the CP ratio is 11, which is much lower than the cat, but its pinwheel density is closer to that of a primate (5.5 pinwheels per mm^2^). Similarly, in the tree shrew the CP ratio is <8, which is even lower than the ferret, but its pinwheel density is higher than recorded in primates (9.4 pinwheels per mm^2^). These inter-species variations probably highlight the influence of multiple factors controlling retinal design and, possibly, cortical map structures in tree shrews and ferrets.

Based on these observations we suggest that the density of cells providing input to the cortex may be related by an all-or-nothing rule that dictates the existence of pinwheel maps versus salt-and-pepper maps. That is, if the CP ratio is <4 the cortex is salt-and-pepper. If the CP ratio is >7 the cortex has a pinwheel structure. A strong bias toward central vision increases the number of retinal inputs to the thalamus, which overexpands V1 and provides the spacing between thalamic axon patches to allow clustering within different cortical domains. Moreover, if a pinwheel map is present, the CP ratio may dictate the density of pinwheels (per mm^2^). Therefore, if we find pinwheel OS maps, we expect to see a gradation in orientation column widths that relates to the CP ratio of the retinal inputs (Figure 6B).

We can use the four species that we have highlighted as future candidates to fill-in the gaps in Figures 2, 3, 6A. (1) The wallaby is known to have a centralized retinal input to the cortex (Figure 5F), with a CP ratio of 20, but its OS map structure is not known. Based on our CP theory, we would expect it to have a pinwheel structure. However, the wallaby has an intermediate number of cells in cortex (3.5 M, Figure 3) that sits between the squirrel and rabbit, which would suggest a salt-and-pepper structure based on the “more cells more structure” theory (Weigand et al., 2017). (2) Evidence from electrophysiological, rather than optical, recording suggests that the agouti may have a salt-and-pepper OS map (Ferreiro, 2018), and it has a low CP ratio of 2.9. We are reasonably confident that the agouti will not have a pinwheel map. (3) The sheep/goat has a mixture of a VS and a small, temporally located “AC” (Shinozaki et al., 2010). They have high retinal cell density and a CP ratio close to 9. Sheep have surprisingly good visual acuity that exceeds that of domestic cats (Clarke and Whitteridge, 1976). We cannot predict with certainty at this point whether the sheep will have pinwheel maps, but we think the evidence favors this prediction, particularly as the electrophysiology data support this notion (Clarke et al., 1976). Also, recall that the sheep has a large cell count in visual cortex, which also supports the idea of a pinwheel map structure (Figure 3). (4) Fruit bats have a centralized retinal input to the cortex, but their CP ratio of 4.5 is low so it will be interesting to know if they have a pinwheel map structure. It is worth noting that while the CP ratio is low in bats, unlike most mammals the drop-off in the periphery is mild, so while the CP ratio is low it is because the peripheral cell density is usually high, compared to the central cell density.

Interestingly, a study was conducted in which nose length and retinal design were compared in dogs (McGreevy et al., 2004). All of the dogs were from the same species (*Canis lupus familiaris*) but were from different man-reared breeds. It was found that dogs with short noses and frontal eyes (e.g. pugs) had a retinal design best described as an AC, while dogs with long noses and lateral eyes (e.g. greyhound) had VSs not dissimilar to rabbits. This finding shows that there may well be a close link between the genetics controlling head configuration and retinal design. Alternatively, being born with eyes that point either frontally or laterally might promote a developmental predisposition toward a particular retinal design. Unfortunately, it was not possible in McGreevy et al.’s (2004) post-mortem study to investigate cortical design in the various dog breeds. While probably impossible to do based on the sensitivity of the community to research on dogs, an obvious experiment to establish the importance of retinal design on cortical organization would be to record cortical maps in dog breeds that have VSs compared to those with an AC. Such an experiment has the natural control that all animals are from the same species.

### Understanding Cortical Maps Through Phylogeny

**FIGURE 7:**
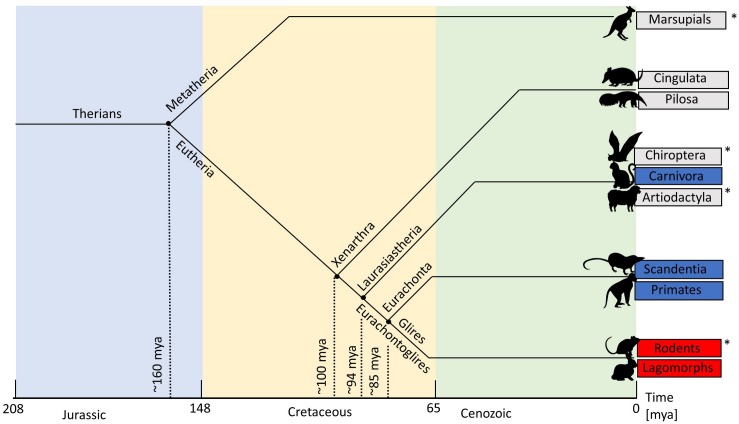
Mammalian phylogenetic tree. Mammalian order names in blue have pinwheels. Order names in red have salt-and-pepper. Order names in gray are those without optical imaging data. Asterisks are those that we suggest should be investigated in future studies: wallaby (Marsupial), fruit bat (Chiroptera), sheep (Artiodactyla), and agouti (Rodents). The dotted lines show where the mammalian clades diverge. MYA, millions of years ago.

Figure 7 provides a visual guide to mammalian phylogeny (Springer et al., 2003; Luo et al., 2011). Virtually everything we know about visual cortex in mammals comes from work on rodents, rabbits, members of the order Carnivora, and primates. These species comprise an extremely small subset (<0.1%) of extant mammals. Rodents and rabbits belong to the mammalian clade Glires and will be referred to collectively by this name (Figure 7). It is not clear whether the Glires have a genetic predisposition not shared by other mammals toward a salt-and-pepper OS map or it is another factor, independent of the phylogenetic relationship, which dictates the map type. From Figure 7, note the large phylogenetic distance between primates (clade, Euarchonta) and the Carnivora (clade, Laurasiatheria), both of which contain species with pinwheel OS maps. In contrast, members of the mammalian clade Glires, which are on the same branch as the primates (Figure 7), have salt-and-pepper maps. As it stands at present, the only clear phylogenetic pattern is that all of the Glires studied so far have a salt-and-pepper map structure, probably including the agouti (Ferreiro, 2018). Given that none of the other mammals studied so far have this pattern, the data suggest that salt-and-pepper maps may be a genetically determined feature of the Glires. However, without adding more species from diverse mammalian orders, this conclusion cannot be verified.

From a phylogenetic standpoint, one very obvious path to take is to study marsupial mammals. Marsupials split away from the placental mammals 130–180 million years ago (Samollow, 2008). Even if just one species has a pinwheel OS map, the genetic code has probably been with the mammals from the beginning (with the proviso that it may have been re-invented through convergent evolution). In the plots presented here we have used the wallaby because its cortex is quite well understood (e.g. Vidyasagar et al., 1992; Ibbotson et al., 1994; Hemmi and Mark, 1998; Ibbotson and Mark, 2003). For reasons already discussed, the rodent agouti and the sheep are also good targets for intrinsic OI. Fruit bats (flying foxes) belong to the mammalian order Chiroptera. These bats have excellent frontal vision similar to that in cats (Figure 7). There has been considerable interest in the primary visual cortex of fruit bats because the structure of the subcortical visual pathways is similar to the “unique” pathways found in primates (Pettigrew, 1986). While the retinotopy of fruit bat visual cortex has been studied and reveals a strong cortical magnification factor in the areas of retinal specialization (Rosa et al., 1993), no measurement of OS maps using OI has occurred.

## Conclusion

Cortical V1 maps in rodents and rabbits do not cluster together cells with similar orientation selectivities. Conversely, all other mammals that have been studied have V1s where cells with similar OS are clustered into highly organized orientation columns. We have pointed out that there are many differences between the eye divergences, retinal designs, visual pathways, and cell counts in V1 between mammalian species. There appears to be a consistent correlation between the CP ratio, which compares retinal cell densities in the central and peripheral retinas, and the presence or absence of OS maps. Other characteristics such as the number of cells in V1 also show reasonable correlations with map design but several species break the clear trend. What is clearly missing in the literature is measurement of OS maps in species that have visual pathway and brain structures that are intermediate between rodents and primates. Therefore, we believe that more comparative observations are essential to constrain future suggestions of OS map formation.

## Author Contributions

MI and YJ wrote the manuscript, produced the figures, developed the concepts discussed, contributed to the manuscript revision, and read and approved the submitted version of the manuscript.

## Conflict of Interest

The authors declare that the research was conducted in the absence of any commercial or financial relationships that could be construed as a potential conflict of interest.
